# Cutaneous skull metastasis from uterine leiomyosarcoma: a case report

**DOI:** 10.1186/1477-7819-7-45

**Published:** 2009-05-11

**Authors:** Nikolaos Barbetakis, Dimitrios Paliouras, Christos Asteriou, Georgios Samanidis, Athanassios Kleontas, Doxakis Anestakis, Kostas Kaplanis, Christodoulos Tsilikas

**Affiliations:** 1 Thoracic Surgery Department, Theagenio Cancer Hospital, A. Simeonidi 2, Thessaloniki, 54007, Greece; 2 General Surgery Department, Theagenio Cancer Hospital, A. Simeonidi 2, Thessaloniki, 54007, Greece; 3 Pathology Department, Theagenio Cancer Hospital, A. Simeonidi 2, Thessaloniki, 54007, Greece; 4 Gynecology Department, Theagenio Cancer Hospital, A. Simeonidi 2, Thessaloniki, 54007, Greece

## Abstract

**Background:**

Cutaneous metastases in the facial region occur in less than 0.5% of patients with metastatic cancer.

**Case presentation:**

A 52-year-old woman who admitted with a lung and a skull skin nodule is presented. She had a known diagnosis of uterine leiomyosarcoma following an extended total hysterectomy two years ago. Excision biopsy of both nodules revealed metastatic disease.

**Conclusion:**

The appearance of a cutaneous nodule in a patient with a history of uterine leiomyosarcoma might indicate a metastatic tumor lesion. Biopsy and immunohistochemistry are essential for correct diagnosis.

## Background

Leiomyosarcoma is a rare malignant neoplasm composed of cells demonstrating smooth muscle differentiation. Uterine leiomysarcoma accounts for 25–36% of uterine sarcoma and 1% of all malignancies and has a poor prognosis due to a high metastatic recurrence rate. They most commonly arise de novo; however, a minority (5%) may be associated with prior irradiation. The peak incidence occurs in the 30–40 age range and reaches a plateau in the middle age. Uterine leiomyosarcoma usually presents with features of vaginal bleeding (77–95%), pelvic pain (33%), uterine enlargement or a palpable pelvic mass (20–50%) [1].

The commonly reported sites of metastasis from leiomysarcoma are the lung, kidney and liver [2]. Spread to the thyroid, brain, bone, skeletal muscle, heart, parotid gland and the oral cavity have also been reported [3-8]. Uterine leiomyosarcoma should be distinguished from benign uterine metastasizing leiomyoma which is diagnosed several years after myomectomy or hysterectomy with most commonly radiographic appearance of slow-growing solitary or multiple lung nodules.

In this report we describe an unusual case of uterine leiomyosarcoma metastasizing to the skull skin.

## Case presentation

A 52-year-old multiparous woman was referred to our hospital in 2006 for post-menopausal abnormal uterine bleeding. She underwent an extended total hysterectomy, bilateral salpingho-oopherectomy and pelvic lymphadenectomy. Tumor cells infiltrated to the uterine serosa and invasion of the tumor cells to the lymphatic vessels was also noted. Immunohistochemistry demonstrated that the tumor cells were positive for a-smooth muscle actin. The patient was diagnosed with uterine leiomyosarcoma (intermediate grade) with positive pelvic lymph nodes. Postoperatively she received further treatment with combination chemotherapy composed of epirubicin, cyclophosphamide and carboplatin for 6 months. She also received radiation therapy with a total of 45 Gy to the pelvis.

**Figure 1:**
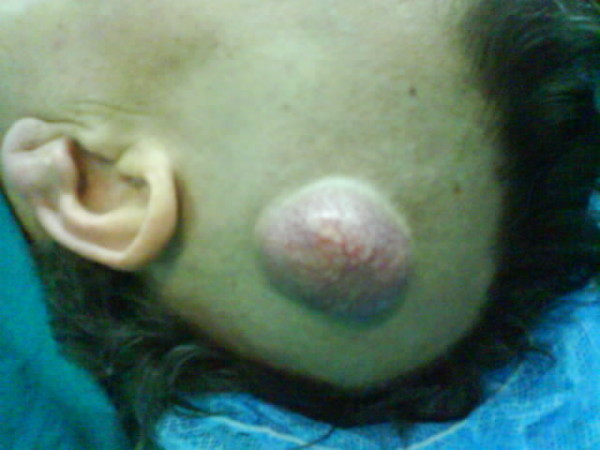
**Clinical examination revealed a nodule on the skull skin**.

**Figure 2:**
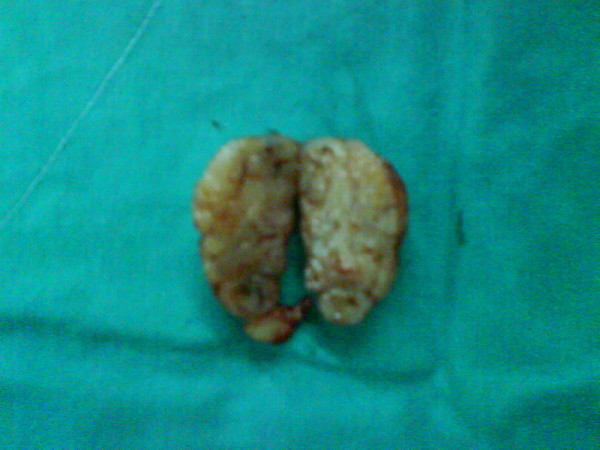
**Macroscopic appearance of the resected nodule**.

**Figure 3:**
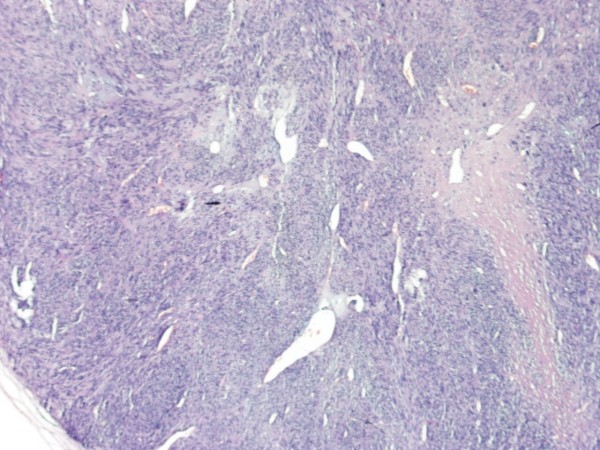
**Pathology of the excised cutaneous nodule consistent with metastatic uterine leiomyosarcoma (cellular eosinophilic spindle cell tumor with nuclear atypia and mitosis) (HE ×40 and ×200)**.

**Figure 4:**
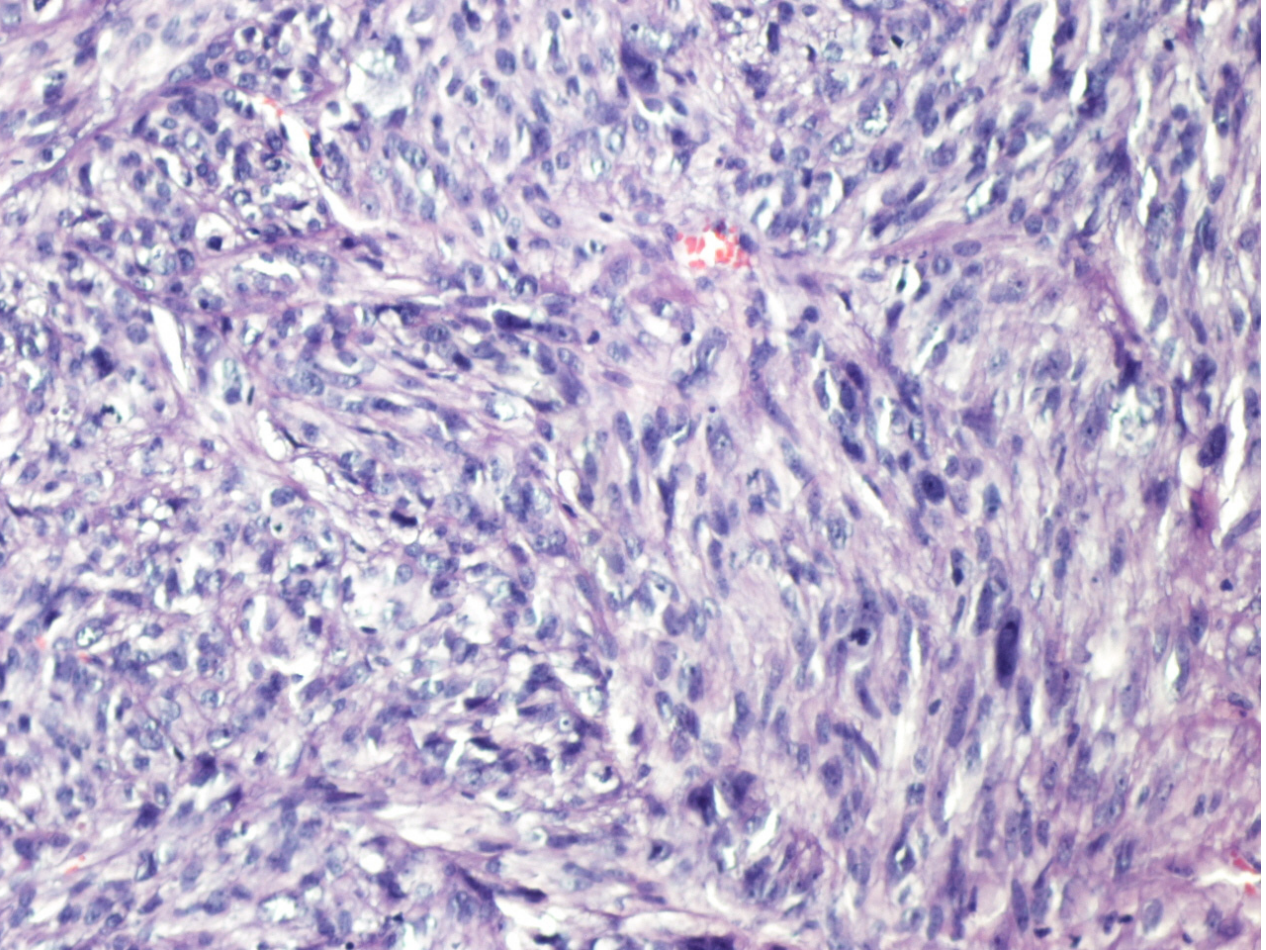
**Pathology of the excised cutaneous nodule consistent with metastatic uterine leiomyosarcoma (cellular eosinophilic spindle cell tumor with nuclear atypia and mitosis) (HE ×40 and ×200)**.

The patient remained asymptomatic for 2 years postoperatively. During regular follow up, computed tomography demonstrated a suspicious lung lesion. Clinical examination also revealed a nodule measuring 4 × 4 cm on the skull skin of the left temporal lobe (Figures 1, 2). Therefore under general anesthesia, she underwent video-assisted thoracic surgery for the pulmonary nodule (wedge resection) and excision biopsy of the cutaneous lesion at the same time. Both of them were diagnosed as metastases from uterine leiomyosarcoma. The excised skin nodule revealed a proliferation of atypical spindle cells with a woven, palisading and rosette-forming pattern surrounded by fibrocollagenous tissue, with a high mitotic ratio (Figures 3, 4). Further immunohistochemical staining was positive for desmin and vimentin and this confirmed the diagnosis. The patient was referred for chemotherapy and 8 months later is still alive but with multiple lung metastases.

## Discussion

Smooth muscle is a component of many tissues and organs. As a result, leiomyosarcoma can arise at almost any anatomic site in the human body. In women, approximately one third of leiomyosarcomas originate in the gastrointestinal tract, particularly the small bowel and colon and another one third are found in the uterus.

Stage, age, tumor size and delivery status of the patient were found to be the most important prognostic factors as regards survival. Interestingly, it seems that higher parity (up to three deliveries) had a negative influence on survival in cases of uterine sarcoma. The relationship between parity and survival in cases of uterine sarcoma should be evaluated more closely in larger series in the future [9].

Extrafascial hysterectomy with pelvic lymph node sampling with or without salpingo-oophorectomy is the surgical gold standard. Debate concerning removal of adnexa and the value of lymph node dissection (LND) is still ongoing [10]. The survival of younger patients with leiomyosarcoma without oophorectomy has been better in one study which is very controversial. The rate of lymph node metastasis has been between 0–47%, and in some studies survival has not been significantly affected as regards LND [11]. The role of adjuvant therapies is controversial. Radiotherapy (RT) seems to improve local control but not survival. Adjuvant chemotherapy (CT) does not decrease the risk of metastatic spread or improve survival. In recurrent uterine sarcomas the response rates in different chemotherapeutic regimens have been between 0–57%. However, the conclusion after a review of the literature was that it is reasonable to offer palliative CT to patients with advanced uterine sarcoma. The effects of hormone therapy in cases of recurrent uterine sarcoma have been assessed in only a few studies [12].

A case of uterine leiomyosarcoma with synchronous lung and cutaneous skull metastasis is presented.

Lung and breast cancers are the commonest epithelial malignancies metastasizing to the skin in men and women respectively. Clinically, cutaneous metastases manifest as nodules, ulceration, cellulitis like lesions, bullae or fibrotic processes [7].

Cutaneous metastases as a first sign of internal malignancy occur infrequently. More commonly, they are early indicators of metastatic disease [8]. Diagnosis may delay several months, unless the skin lesion grows rapidly or other sites such as the lung or liver affected by tumor spread. In our case, the cutaneous metastasis was diagnosed simultaneously with the lung lesion.

Uterine leiomyosarcoma has a strong metastatic potential to distant sites, because of its aggressiveness and propensity for hematogenous spread. Cutaneous metastasis although rare indicates tumor relapse. Early detection requires high index of suspicion. Therefore, close inspection of new skin lesions in patients with history of malignancy is imperative and diagnostic biopsy is essential.

## Consent

Written informed consent was obtained from the patient for publication of this case report and accompanying images. A copy of the written consent is available for review by the Editor-in-Chief of this journal.

## Competing interests

The authors declare that they have no competing interests.

## Authors' contributions

NB, DP, CA, GS, AK, DA and KK took part in the care of the patient and contributed equally in carrying out the medical literature search and preparation of the manuscript. CT participated in the care of the patient and had the supervision of this report. All authors approved the final manuscript.
